# Residual Infestation and Recolonization during Urban *Triatoma infestans* Bug Control Campaign, Peru^1^

**DOI:** 10.3201/eid2012.131820

**Published:** 2014-12

**Authors:** Corentin M. Barbu, Alison M. Buttenheim, Maria-Luz Hancco Pumahuanca, Javier E. Quintanilla Calderón, Renzo Salazar, Malwina Carrión, Andy Catacora Rospigliossi, Fernando S. Malaga Chavez, Karina Oppe Alvarez, Juan Cornejo del Carpio, César Náquira, Michael Z. Levy

**Affiliations:** University of Pennsylvania, Philadelphia, Pennsylvania, USA (C.M. Barbu, A.M. Buttenheim, M.Z. Levy);; Universidad Peruana Cayetano Heredia, Arequipa, Peru (M.-L. Hancco Pumahuanca, J.E. Quintanilla Calderón, R. Salazar, M. Carrión, C. Náquira);; Red de Salud Aequipa Caylloma, Arequipa (A. Catacora Rospigliossi);; Dirección Regional del Ministerio de Salud, Arequipa (F.S. Malaga Chavez, K. Oppe Alvarez, J. Cornejo del Carpio)

**Keywords:** Chagas disease, Trypanosoma cruzi, Triatoma infestans, bugs, vector, infestation, recolonization, urban vector control, campaign, Peru

## Abstract

Chagas disease vector control campaigns are being conducted in Latin America, but little is known about medium-term or long-term effectiveness of these efforts, especially in urban areas. After analyzing entomologic data for 56,491 households during the treatment phase of a *Triatoma infestans* bug control campaign in Arequipa, Peru, during 2003–2011, we estimated that 97.1% of residual infestations are attributable to untreated households. Multivariate models for the surveillance phase of the campaign obtained during 2009–2012 confirm that nonparticipation in the initial treatment phase is a major risk factor (odds ratio [OR] 21.5, 95% CI 3.35–138). Infestation during surveillance also increased over time (OR 1.55, 95% CI 1.15–2.09 per year). In addition, we observed a negative interaction between nonparticipation and time (OR 0.73, 95% CI 0.53–0.99), suggesting that recolonization by vectors progressively dilutes risk associated with nonparticipation. Although the treatment phase was effective, recolonization in untreated households threatens the long-term success of vector control.

## Abstract 2

Recolonization from untreated households is a serious threat to long-term vector control.

Chagas disease, an often deadly disease widespread in the Americas, is caused by the protozoan parasite *Trypanosoma cruzi* (*1**,**2*) and transmitted by hematophageous triatomine insects (*3*). In southern South America *Triatoma infestans* bugs are the primary vector (*2*). In 1991, the nations of this region created the Southern Cone Initiative to coordinate control efforts against *T. infestans* bugs. During the first decade of this initiative, 2.5 million households were treated with insecticide (*4*), which led to disruption of transmission of *T. cruzi* by *T. infestans* bugs in several countries and states (*2*). However, vector control efforts have at times failed unexpectedly, and repeatedly in some areas (*5**,**6*).

Across most of their range, *T. infestans* insects are found predominantly in rural areas (*2*). However, the vector has become an urban problem in Arequipa, Peru, a city of 850,000 inhabitants (*7**–**9*) where infected vectors have been observed since 1952 (*10*). Since 2003, municipal authorities and the regional ministry of health, in collaboration with the Pan American Health Organization, have worked to eliminate the vector from this city. The challenges to elimination in an urban area potentially differ from those in rural settings. Urban households have smaller peridomestic areas, fewer sources of blood, and fewer hiding places for the vector, thus mitigating some of the difficulties encountered in rural environments (*7**,**11**–**13*). However, whereas participation in control campaigns in rural areas is typically high (*5**,**7*), more affluent urban populations (*14*) might be more reluctant to participate (*15*). Thus, household level control might be easier in an urban household than in a rural household. However, at the community level, sustained control in an urban community might be more difficult.

We explored this hypothesis by using data obtained in Arequipa during the initial treatment phase or attack phase of the vector campaign and during the subsequent surveillance phase after insecticide application. We evaluated the effectiveness of the treatment phase in 3 ways. First, we estimated the reduction in the infestation prevalence resulting from the 2 insecticide applications of the treatment phase. Second, we modeled recolonization (the colonization of new households after the initial treatment) as a function of treatment phase factors. Third, during the surveillance phase, we tested insects captured from households treated during the treatment phase for resistance to insecticide. We discuss converging results of these approaches in terms of their implications for continued efforts of the control campaign in Arequipa and, more generally, for design of strategies to control Chagas disease vectors in urban environments.

## Materials and Methods

### Campaign

**Figure 1:**
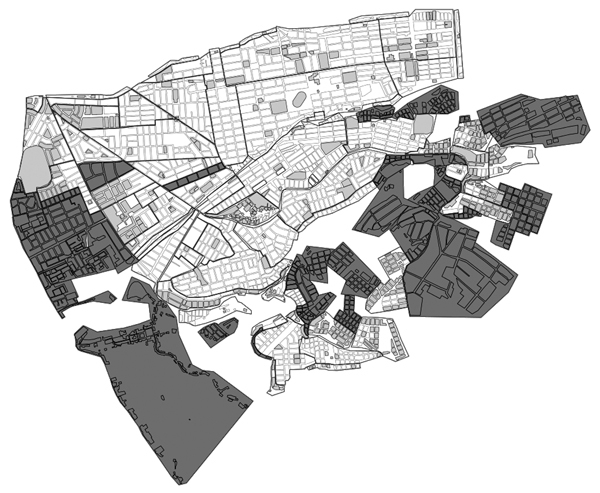
Areas targeted for Chagas disease vector control in the Paucarpata District, Arequipa, Peru. Small units are city blocks and large units are localities. Dark gray indicates localities not infested; light gray indicates areas targeted; and medium gray indicates nontargeted city blocks within infested localities.

The vector control campaign in Arequipa proceeded through the city district by district. Districts, local administrative subdivisions of the region, comprise 3,000–28,000 households. Within each district, treatment is organized on a locality level; localities vary in size from 50 to 2,000 households. Preliminary inspections in the months preceding the treatment phase identified localities (or city blocks within a locality) as sufficiently infested to warrant treatment (Figure 1; Table 1). However, the results of these preliminary inspections at the household level are available only for the most recent surveys.

**Table 1:** Targeted areas within districts and localities and distribution of reports, inspections, and uncovered infestation during surveillance phase of a Chagas disease vector control program, Arequipa, Peru, 2003–2011*

Nested units	City blocks	Households	Households reporting infestations	Households inspected	Households inspected and found infested
Participating districts, n = 8	5,955	79,972	301	785	145
Infested localities, n = 256	4,755	67,218	258	714	133
Targeted areas	3,727	56,491	225†	613	116‡

*Reporting and inspected households during surveillance are counted from January 2009 through end of December 2012.  †Households in targeted city blocks have a similar rate of report and risk of being positive during the surveillance phase as other households in their districts: odds ratio 1.12, p = 0.45; and 0.94, p = 0.77, controlling for diversity between districts. ‡Among these households, 77 were reporting households (35% of the 219 inspected reporting households).

After inspections, localities entered the treatment phase. Health promoters and vector control specialists visited every household in the targeted areas to apply insecticide (http://www.spatcontrol.net/articles/Barbu2014/suppMet.pdf) to all domestic and peridomestic structures. These visits occurred twice at 6-month intervals. All households in targeted areas were asked to participate in this phase. As houses were treated, trained personnel collected *T. infestans* specimens flushed out of their refuges by the insecticide. Six months after the second treatment, localities entered the surveillance phase, a community-based effort to identify residual and returning vector populations. All households in the district were eligible to participate in the surveillance phase, even if they were not targeted for the treatment phase. In the surveillance phase, households reported infestation, and campaign staff conducted inspections and treated areas in and around reporting households. 

Inhabitants were asked to bring any *T. infestans* insects found in their households to community health workers or health posts located throughout the city. Trained entomologic technicians systematically searched for and collected *T. infestans* insects in indoor and peridomestic areas of reporting households and their immediate neighbors (1 person-hour search per household) by using aerosol spray containing tetramethrin (Mata Moscas 0.15% tetramethrin; Sapolio, Doral, FL, USA), which has a strong flushing out effect on triatomine insects but does not kill them. All captured insects were counted, staged, sexed, and microscopically examined for *T. cruzi* as described previously (*7*). Infested households and their immediate neighbors were treated with insecticide as in the treatment phase.

For all phases of the campaign, a household with at least 1 observed *T. infestans* bug of any developmental stage was considered infested (eggs were not collected or reported) (http://www.spatcontrol.net/articles/Barbu2014/suppMet.pdf). For all control activities, a unique identifier code, participation (0/1), and infestation status (0/1) of each household were recorded. We mapped the exact positions of households and city blocks by using satellite imagery in Google Earth (*16*) and field maps drawn by Ministry of Health personnel. Household geographic unique identifier codes, coordinates, participation status, and *T. infestans* bug infestation status were stored for subsequent analysis (*17*).

### Sample

During 2004–2011, eight urban districts participated in the treatment phase of the campaign (Jacobo Hunter, José Luis Bustamente y Rivero, La Joya, Paucarpata, Sachaca, Uchumayo, Tiabaya, and Socabaya); they encompassed 356 localities. These localities correspond to an urban-to-periurban environment as described by Delgado et al. (*17*). These districts comprised a total of 79,972 households in 5,955 city blocks (Table 1; Figure 1). However, 2,228 (37.4%) city blocks in these districts were not included in the treatment phase because no vectors were observed during preliminary inspections. The remaining 3,727 city blocks that were included contained 56,491 households. Because we aimed to find an association between treatment phase and infestation during the surveillance phase, we restricted our analysis to these city blocks and households. We report results of the surveillance phase inspections over a 4-year period (2009–2012) during which our study team collaborated with the Peru Ministry of Health to monitor infestation. Surveillance is still ongoing in these areas.

### Statistical Analysis

#### Modeling Effectiveness of Treatment Phase and Residual Infestation

We included 3 parameters in our model of treatment phase effectiveness. The first parameter was *c,* the probability of clearing treated households of *T. infestans* bug colonies with 1 treatment. The second was *s,* the sensitivity of detection, defined as the probability that trained entomologic technicians performing house treatment would observe infestation when it is present. The third was *_I/II_*, the true number of infested households immediately before any treatment.

To estimate these parameters, we compared the infestation observed during the first and second treatments of the initial treatment phase in the 35,207 households that accepted both treatments (Table 2). Households were unlikely to become infested between the 2 treatments: the treatments were separated by only 6 months, the overall infestation is severely reduced by the first treatment, and treated households are protected by the residual effect of the insecticide for several months (*18*). Assuming that households did not become infested between the 2 treatments, we jointly estimated *c*, *s,* and *n_I/II_* by modeling the observed infestation as a system of 3 equations (observed infested twice, infested only treatment I, and infested only during treatment II) (Table 3) that we solved analytically. In addition, we considered this model from a stochastic perspective to compute CIs for our estimates; further details are available online (http://www.spatcontrol.net/articles/Barbu2014/suppMet.pdf).

**Table 2:** Estimations of initial and residual infestation for treatment phase of a Chagas disease vector control program, Arequipa, Peru, 2003–2011*

Treatment received	No. households	Initial prevalence, %			Estimated residual infestation	
		Observed	Estimated		Prevalence, %	No. (%) infested households
I and II	35,207	20.1	35.6		0.006	2 (0.3)
I only	7,521	7.0	12.2		0.16	14 (2.1)
II only	4,169	4.0	6.9		0.09	4 (0.5)
Not treated	9,594	ND	6.9†		6.9	666 (97.1)
Total	56,491	16.2	25.8		1.2	686 (100)

*Estimates are calculated by using equations in Table 3 (estimated sensitivity of inspectors p = 57% [range 46%–66%] and probability of clearing households of infestation through 1 treatment c = 98.7% [range 98.4%–98.9%]). ND, no data.  †Extrapolation of the prevalence in households participating only in the second treatment to households that were never treated.

**Table 3:** Household level model of observation, initial infestation, treatment, and residual infestation during treatment phase of a Chagas disease vector campaign, Arequipa, Peru, 2003–2011*

Treatment received	Observed infestation	Estimated initial prevalence	Estimated residual prevalence
I and II		*p_I/II_* =* n_I/II_/T_I//II_*	*r_I/II_* =* p_I/II_* × (1* – c*)^2^
		
		
I only	*O_I+IIØ_* =* n_I_* ×* s*	*p_I_* =* n_I_/T_I_*	*r_I_* =* p_I_* × (1* – c*)
II only	*O_IØII+_* =* n_II_* ×* s*	*p_II_* =* n_II_/T_II_*	*r_II_* =* p_II_* × (1* – c*)
None	Not observed	*p_Ø_* =* p_II_*	*r_Ø_* =* p_Ø_*

*Upper case letters refer to observed quantities. Lower case letters refer to estimated quantities. *O_IxIIy_*, number of infested households observed in the first (*Ix*) and second (*IIy*) step of the treatment phase, with *x* and *y* taking the following values: *∅*, no treatment and infestation could not be observed; +, treated and observed infested; –, treated and observed noninfested. For *n_z_*, *p_z_*, *T_z_*, and *r_z_*, the subscript z corresponds to the participation in treatments: *I*, only first; *II*, only second; *I/II*, both; *n_z_*, estimated number of infested households; *p_z_*, estimated initial prevalence of infestation; *T_z_*, total number of households; *r_z_*, estimated residual prevalence of infestation post treatment phase; *s*, sensitivity of technicians performing treatment to household infestation; *c*, probability of clearing infestation when treated. Further details and the solved system of equations have been provided by the authors (http://www.spatcontrol.net/articles/Barbu2014/suppMet.pdf).

We extrapolated infestation prevalence before treatment in households treated only once by using the estimated sensitivity of the infestation detection (*s*) (Table 2). For households that were never treated with insecticide, and on the basis of other data (http://www.spatcontrol.net/articles/Barbu2014/suppMet.pdf), we used the prevalence found in households that were only treated during the second insecticide treatment.

Finally, we estimated residual infestation (number of households still infested after the treatment phase), assuming that the effects of the first and second insecticide spraying of the treatment phase are comparable and independent. We applied the clearing rate estimated during the first treatment, *c*, to the estimated initial infestation once or twice according to the number of treatments (Table 3). We examine the validity of this assumption below; other details are available online (http://www.spatcontrol.net/articles/Barbu2014/suppMet.pdf).

#### Logistic Mixed Effect Models of Infestation during Surveillance

We used univariate and multivariate mixed effect logistic regressions (*19*) to describe the correlations between treatment phase and surveillance observations. Unless otherwise noted, we used a random effect term to control for potential similarity of households in a locality (*20**,**21*).

Our first multivariate logistic model describes the probability that households targeted during the treatment phase were infested at least once during the surveillance phase as a function of observed infestation of the household during the treatment phase, participation of the household in the treatment phase, and number of years the household had been under surveillance as of January 1, 2013. We also considered the interaction between time and the other risk factors. The outcome assessed in the first model consists of 2 processes: selection of inspected households (reporting households and their neighbors) and individual infestation status of inspected households. We investigated these processes separately in the second and third logistic models.

In our second logistic model, we estimated the probability that an infestation report was generated on a city block given the number of infested and participating households on the block during the treatment phase. In our third logistic model, we estimated the probability of infestation among inspected households given the household infestation and participation status during the treatment phase; we used a random effect term to control for potential similarity of households around a household that generated a report. A total of 225 reports were generated. Because some households had been inspected multiple times during the study, we limited our sample to the first inspection following the treatment phase. Because sensitivity and specificity of our surveillance program rely on community reports, we assessed their reliability (http://www.spatcontrol.net/articles/Barbu2014/suppMet.pdf). We estimated the goodness-of-fit for these models by using Nagelkerke pseudo R^2^ (*22*). All computations were performed by using R (*23*).

### Evaluation of Susceptibility of Residual Populations to Pyrethroid Insecticide

During the surveillance phase, we collected triatomine insects from infested households that had been treated during the treatment phase to evaluate resistance to deltamethrin. We followed guidelines for wall bioassays of the World Health Organization/Special Programme for Research and Training in Tropical Diseases (*24*). In brief, we applied 5% deltamethrin suspension concentrate (K-Othrine SC; Bayer, Leverkuesen, Germany) with an X-Pert compression sprayer (Hudson Manufacturing Co., Chicago, IL, USA) at the target dose of 25 mg/m^2^ to a cemented wall and allowed it to dry for 24 h. From each household, we placed 10 F_1_ progeny fifth instar nymphs in a petri dish on the wall for 72 h. We evaluated the status of the insects 3 days after the exposure. The insects used in the bioassay had molted 15–20 days before the experiment, and had fasted for 7 days. Throughout the experiment, insects were kept under ambient conditions in our field laboratory (19–28°C, humidity 24%–51%).

## Results

### Treatment Phase Effectiveness and Estimated Residual Infestation

Among the 56,491 households targeted for the initial treatment phase in Arequipa, 46,897 (83%) participated in at least 1 of the treatments. A total of 35,207 (62%) households targeted for the treatment phase were treated twice, 11,690 (21%) were treated only once, and 9,594 (17%) were not treated in either step of the treatment phase (Table 2). Participation in the 2 treatments showed a strong correlation (odds ratio [OR] 1.56, 95% CI 1.54–1.57).

Our model comparing the infestation observed during the first and second insecticide treatments suggested that a single treatment was successful in 98.7% (95% CI 98.4%–98.9%) of infested households (Table 2) (http://www.spatcontrol.net/articles/Barbu2014/suppMet.pdf). Among households treated twice, estimated prevalence of infestation decreased from 35.6% before the treatment phase to 0.006% after the second treatment. Estimated initial prevalence of infestation in households participating in a single treatment (range 6.9%–12.2%) was lower than that of households that participated twice (35.6%), which suggested a strong correlation between infestation status and participation. Estimated probability of detecting infestation in an infested household during a treatment (*s*) was 57% (95% CI 0.46–0.66) (Table 2) (http://www.spatcontrol.net/articles/Barbu2014/suppMet.pdf), which is comparable to previous estimates obtained by using other methods (*25*).

Similar results were found for the district of Mariano Melgar (treated during 2011–2012) by using a difference-in-difference approach (http://www.spatcontrol.net/articles/Barbu2014/suppMet.pdf). We observed a strong reduction in infestation attributable to the treatment (OR 0.02, 95% CI 0.006–0.09]) and a strong effect of infestation on participation in the treatment (OR 4.4, 95% CI 3.5–4.5). These 2 analyses suggest that after the initial treatment phase, households that received no treatment represent >90% of infested households.

### Surveillance Phase Infestations and Their Relationship to Treatment Phase

During 2009–2012, surveillance authorities received 225 reports of vector infestations within the area targeted by the treatment phase (Table 1). A total of 613 houses (including reporting houses and their immediate neighbors) were inspected during the surveillance phase. Of these 613 households, 116 (19%) were infested (http://www.spatcontrol.net/articles/Barbu2014/suppMet.pdf). Of the 116 infested houses, 29 (25%) had never been treated, 18 (16%) had been treated only once, and most (69, 59%), had been treated twice, which indicated that recolonization can occur in treated households. Among the 87 households found to be infested after treatment, most (49, 56%), had no history of infestation before treatment. No houses were observed to be continuously infested during the 2 steps of the treatment and during the surveillance phase (http://www.spatcontrol.net/articles/Barbu2014/suppMet.pdf), which suggested that treatment was highly effective.

**Figure 2:**
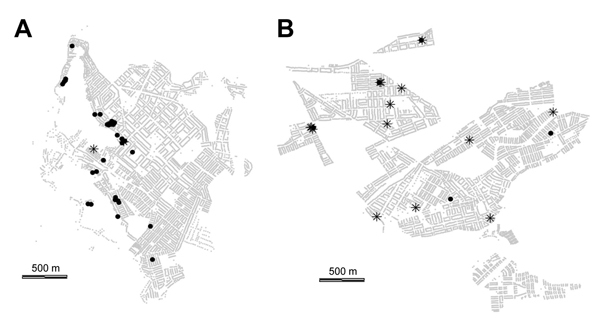
Infestation during the surveillance phase of a Chagas disease vector control program shown by history of treatment during the treatment phase for A) Jacobo Hunter District (treatment phase during 2003–2005) and B) Paucarpata District (treatment phase during 2006–2009, Arequipa, Peru. Stars indicate households infested during surveillance phase and not treated during treatment phase; black circles indicate households infested during surveillance phase but treated during the treatment phase; and light gray dots indicate other households (their alignment produces what appears as the background pattern of streets).

Maps of infestation as observed during the surveillance phase and treatment histories of 2 representative districts treated during 2003–2005 and 2007–2009, respectively, show different patterns of vector presence (Figure 2). In the more recently treated district, most households (13/18, 72%) that were found to be infested during the surveillance phase had not been treated during the treatment phase; in the district treated earlier, infestation was present mainly in treated households (30/32, 94%). In the more recently treated area, infestation of a treated household was usually (3/5, 60%) associated with at least 1 neighboring nontreated infested household, whereas in the area treated earlier, such infestations were rare; only 3 (9%) of 34 were associated with neighboring nontreated households.

Among 116 households identified as infested by the surveillance program in areas targeted by the treatment phase, we found 16 households with *T. cruzi–*infected *T. infestans* bugs. These 16 households were in 5 districts and showed a strong spatial positive autocorrelation <50 m (http://www.spatcontrol.net/articles/Barbu2014/suppMet.pdf). Most (75%) of these households were treated at least once during the treatment phase.

### Risk Factors for Infestation during Surveillance

Our first logistic model (Table 4) suggests that nonparticipation in, and infestation before, the treatment phase affected the observation of infestation in households during the surveillance phase. Nonparticipation and initial infestation were associated with the probability of inspection through generation of reports at the city block level (Table 5) and with the probability of infestation among inspected households (Table 5). The strong effect of time in the surveillance phase on infestation (Tables 4, 5) suggests a dynamic pattern of recolonization. The negative interaction between time since treatment and nonparticipation in the treatment phase suggests recolonization from nonparticipating households to their neighbors. The recolonization progressively dilutes the association between infestation and untreated houses. The lack of a strong interaction between time and nonparticipation at the city block level suggests less dispersal of the vectors between city blocks. Infestation before treatment is also a strong predictor of infestation during surveillance. The persistence of this association over time, combined with the progression of infestation, suggests that previous infestation is a risk factor for recolonization. This interpretation is also consistent with equally likely alternative models (http://www.spatcontrol.net/articles/Barbu2014/suppMet.pdf).

**Table 4:** Predictors of infestation among all households by *Triatoma infestans* bugs during the surveillance phase (2009–2012) in areas of Arequipa, Peru, treated during 2003–2011)*

Predictor	OR	2.5% CI	97.5% CI
Intercept	2.1 × 10^–5^†	3.2 × 10^–6^	1.4 × 10^–4^
Infested during treatment phase	3.21†	1.96	5.24
Untreated during treatment phase	21.5‡	3.35	138
Time, y	1.55‡	1.15	2.09

*Time corresponds to the number of years after the end of the initial treatment phase in the locality as of January 1, 2013. A locality level random effect is included. CIs assume asymptotic normality. n = 56,491 (116 infested), Nagelkerke pseudo-R^2 ^= 0.16. OR, odds ratio. †p<0.001.  ‡p<0.01.

**Table 5:** Predictors of confirmed reports at the city block level and predictors of observed infestation by *Triatoma infestans* bugs among infested households during surveillance phase of vector control campaign in urban districts of Arequipa, Peru, 2009–2012*

Confirmed reports at city blocks level					Infestation among inspected households			
Predictor	OR	2.5% CI	97.5% CI		Predictor	OR	2.5% CI	97.5% CI
Intercept	8.2 × 10^–5^†	4.5 × 10^–6^	1.5 × 10^–3^		Intercept	0.08†	0.05	0.12
Log(no. infested + 1)	2.08†	1.42	3.04		Infested	2.24‡	1.28	3.91
Time, y	1.69§	1.10	2.58		–	–	–	–
Log(no. untreated + 1)	4.85§	1.34	17.49		Untreated	5.88†	3.18	10.88
Time × log(no. untreated + 1)	0.82¶	0.66	1.02		–	–	–	–

*For confirmed reports at city blocks level, outcome is the existence of ≥1 confirmed report on the city block: reports leading to observing ≥1 infested household on the city block in subsequent inspections. Infested is number of households observed to be infested at least once during the treatment phase. Untreated is number of households not treated in the treatment phase in the city block. Time corresponds to number of years as of 2012 since the end of the initial treatment phase for the locality. A locality level random effect is included for the city block level analysis. OR, odds ratio. N = 3,727 city blocks (80 with confirmed reports) Nagelkerke pseudo-R^2^ = 0.71. For infestation among inspected households, outcome is the infestation status during the first inspection during the surveillance phase. Infested is households infested in ≥1 inspection of the treatment phase. Untreated is households not treated during the treatment phase. We used a random effect on inspections batches around a reporting house. –, because all houses in a batch are inspected at the same time, time is not included as a predictor. CIs are + values and assume asymptotic normality. n = 613 inspected (102 infested). Nagelkerke pseudo-R^2 ^= 0.91. †p<0.001.  ‡p<0.01.  §p<0.05.  ¶p<0.1.

### Insecticide Bioassays

We tested 21 vector populations from 12 localities from 6 of the 8 study districts for deltamethrin resistance. All insects exposed were found dead as defined by the World Health Organization (*24*) three days after the end of the exposure, which suggested that residual populations did not have complete resistance to the insecticide.

## Discussion

Our results provide a consistent picture of the control of *T. infestans* bugs in Arequipa. After the treatment phase, infesting insects were successfully eliminated in nearly all participating households. Immediately thereafter, most infestations were attributable to the households that never participated in the campaign. In the years after treatment, these untreated households served as sources of insects that recolonized their neighbors. Recolonization disproportionately affects households that were infested before control activities, probably because of the continued presence of risk factors for infestation (e.g., poor quality of buildings) (*7**,**11**,**12**,**26*).

Similar studies in rural areas emphasize the role of residual populations of vectors in the recolonization of communities following vector control (*26**–**30*). Recolonization in urban areas was perhaps predictable because insects rapidly move between households (*25**,**31*). However, the problem of residual vector populations is different in our urban study area. In the city, residual infestation is caused mainly by lack of participation; in rural settings, by contrast, participation rates are typically high, and residual vector populations generally originate from locations that are difficult to treat (*26**–**30*).

Recently, insecticide-resistant populations of *T. infestans* bugs have been detected in the Gran Chaco of Argentina, Bolivia, and Paraguay, and many authors have highlighted the possibility that these resistant populations might contribute to the failure of vector control efforts (*32**–**34*). Persistent populations have also been observed following the treatment phase in Cochabamba, Bolivia; however, the reason for this persistence is not understood (*35*). Our study suggests that resistance is not a major cause of residual infestation in Arequipa. This result is reassuring, but partial resistance to insecticides might still be present (*36*).

There are various limitations to our study. Our analysis of the treatment phase assumes independence and similar effectiveness of the 2 treatments. If we relax this assumption, 78% of residual infestations would still have occurred in untreated households, only marginally decreasing the overall effectiveness of the treatment phase (http://www.spatcontrol.net/articles/Barbu2014/suppMet.pdf). In practice, no household was observed to be infested during both treatments of the initial treatment phase and surveillance inspections, which suggested effectiveness of the second treatment. In addition, because there is a strong correlation between infestation and participation (*15**,**21*), households that never participated might have initially a lower prevalence of infestation than those that participated once, in contrast to the equal prevalence we assumed in our analysis. Assuming an overly conservative 5-fold lower prevalence among never-treated houses relative to once-treated households, the never-treated households would still represent >85% of the residual infestation after the treatment phase (http://www.spatcontrol.net/articles/Barbu2014/suppMet.pdf). To date, we have not observed any evidence of clustering of infestation during the surveillance phase around nontargeted areas. However, the presence of these areas, similar to that of untreated households in targeted areas, poses a potential risk for recolonization; consequently, surveillance efforts should continue to include these areas.

Despite the efficiency of treatment, we observed a strong effect of previous infestation. The permanence of factors known to favor infestation (e.g., presence of guinea pigs and building materials) might explain the higher recolonization rate. However, we could not quantify the contribution of different factors to this risk. We also did not assess the risk for transmission of the parasite. Although it appears that treated areas of the city are not under an immediate threat of transmission, a recolonization of the city would create fertile ground for reintroduction of *T. cruzi* infections. The observation during the surveillance phase of *T. cruzi* infection in several households that participated in the initial treatment phase highlights this risk. Finally, although this study covered only 1 city, it encompasses a large part of what comprises one of the main infested urban areas in Latin America and is characterized by diverse landscapes ranging from semi-rural/periurban (*37*) to urban (*25*).

For long-term control of *T. infestans* bugs, the rate of detection and elimination of vector populations in surveillance must exceed that of recolonization. The preponderant role of untreated households in maintaining infestation suggests that when a household reports the presence of a vector during surveillance, we should identify houses in the vicinity that did not participate in the control campaign and target them for inspections and treatment. The higher risk for recolonization of previously infested households also suggests that active surveillance of initially highly infested areas might be useful.

After confirmed absence of vector-borne transmission of *T. cruzi* in Chile, Uruguay, Brazil, eastern Paraguay, and parts of Argentina, southern Peru and the Gran Chaco region represent the last bastions of domestic infestation by *T. infestans* bugs (*2**,**38**,**39*). It is too early to tell whether long-term control will be achieved more or less easily in cities than in rural areas. However, our results confirm our core hypothesis: lower participation in cities such as Arequipa is the main obstacle to the effectiveness of treatment. Identification of nonparticipating households as the main reservoir for residual infestation after the treatment phase opens new options for long-term sustainable control.
